# The proteasomal switch as a kinetic battleground for influenza A virus pathogenesis

**DOI:** 10.1080/22221751.2026.2755221

**Published:** 2026-10-26

**Authors:** Sourabh Soni, Vandana Anang, Pankaj Kumar, Anteneh Mehari Tizazu, Eldana G. Arega, Richard S. Nho, Ana L. Mora, Mauricio Rojas, Jing Zhao, Yutong Zhao, Joseph S. Bednash, Jeffrey C. Horowitz, Jacob S. Yount, Yohannes A. Mebratu

**Affiliations:** a Department of Internal Medicine, Division of Pulmonary, Critical Care and Sleep Medicine, College of Medicine, The Ohio State University Wexner Medical Center, Davis Heart and Lung Institute, Columbus, OH, USA; b Department of Physiology and Cell Biology, College of Medicine, The Ohio State University Wexner Medical Center, Davis Heart and Lung Institute, Columbus, OH, USA; c Department of Microbial Infection and Immunity, College of Medicine, The Ohio State University Wexner Medical Center, Columbus, OH, USA

**Keywords:** Influenza A virus (IAV), immunoproteasome (iP), proteasomal switch, antigen presentation, viral subversion, inflammaging, host-directed therapy

## Abstract

Influenza A virus (IAV) infection initiates a high-stakes competition for proteostatic control. While the host reconfigures its degradative machinery for defense, the virus simultaneously hijacks host proteostatic and degradative pathways to facilitate replication and evade immunity. Central to this conflict is the proteasomal switch, a process in which constitutive proteasome subunits (β1, β2, β5) are replaced by their inducible counterparts (β1i, β2i, β5i) to form the immunoproteasome (iP). Although iP is classically recognized for optimizing MHC class I antigen presentation, emerging evidence reveals more nuanced roles in IAV infections. This review synthesizes current knowledge on iP as an effector of host defense and target for viral subversion. We synthesize emerging evidence and propose working models detailing how IAV proteins may selectively interfere with proteasomal subunits to stabilize pro-viral factors. Furthermore, we examine the hypothesis that the iP functions alongside broader ubiquitin-proteasome system (UPS), endoplasmic reticulum-associated degradation (ERAD), and autophagic pathways as a proteostatic buffer to mitigate proteotoxic and oxidative stress in the infected epithelium. By evaluating how age-related proteostatic decay and viral subversion could impair cellular clearance machinery, we provide a conceptual framework for understanding how proteasomal dysregulation contributes to acute lung injury and the chronic inflammatory state termed inflammaging. Finally, we propose a hypothesis-generating, biphasic framework for timing-dependent proteasomal modulation, contrasting preclinically supported late-stage β5i inhibition against the theoretical concept of early-stage activation to highlight translational hurdles in severe influenza.

## 1. Introduction

### 1.1. The race for proteostatic control in IAV infection

Influenza A virus (IAV) remains an important global health threat, characterized by seasonal epidemics that cause up to 650,000 deaths annually and sporadic pandemics with heightened mortality [1–3]. Clinical severity of influenza reflects a race between viral replication and the host immune response. Upon entry into the respiratory epithelium, IAV hijacks host dependency factors to reach critical titers before an effective antiviral state is established [4,5]. Host cells respond to infection by producing and secreting type I and III interferons (IFN) that in turn trigger hundreds of interferon-stimulated genes (ISGs) in surrounding cells [6]. A pivotal, yet underappreciated, component of this defense is the proteasomal switch, the transition from constitutive proteasomes (cP) to immunoproteasomes (iP) [7,8]. This switch acts as a critical proteostatic buffer and reshapes the cellular antigenic landscape for CD8^+^ T cell recognition [9–11]. Additionally, the proteasomal switch and iP have non-canonical roles in antiviral defense beyond antigenic peptide generation, which are the primary focus of this review.

### 1.2. Ubiquitin-proteasome system (UPS) as a master regulator of proteostasis

Cellular homeostasis relies on the ubiquitin-proteasome system (UPS), the primary pathway for the selective degradation of damaged, misfolded, or short-lived proteins [12,13]. This ATP-dependent process utilizes a hierarchical enzymatic cascade (E1, E2, and E3 ligases), where E3 ligases serve as the critical determinants of substrate specificity [14]. The effector of this system is the 26S proteasome, comprised of a 19S regulatory particle and a 20S catalytic core. The catalytic core contains three primary proteolytic subunits: β1 (caspase-like, cleaving after acidic residues), β2 (trypsin-like, cleaving after basic residues), and β5 (chymotrypsin-like, cleaving after hydrophobic residues) [15]. The transition from cP to iP is a tightly regulated assembly process involving *de novo* synthesis and incorporation of inducible β subunits (β1i (LMP2), β2i (MECL-1), and β5i (LMP7)) in response to pro-inflammatory cytokines, particularly IFN-γ. During this proteasome switch, infection, inflammation, oxidative stress, and other environmental stressors promote replacement of constitutive subunits with their inducible counterparts [16–18]. This transition represents a qualitative reorganization of the cellular degradation machinery rather than a simple increase in protein expression. The resulting iP possesses altered cleavage preferences that favor peptides with hydrophobic C-termini, which bind with higher affinity to major histocompatibility complex class I (MHC-I) molecules [9]. Quantitative proteomics indicate that the MHC-I-presented peptide repertoire changes by nearly 50% in the absence of iP [19].

A critical biochemical trade-off occurs during the transition from the constitutive β5 subunit to its inducible counterpart, β5i. While β5i is enzymatically optimized for generating MHC-I peptides, the constitutive β5 subunit possesses a distinct catalytic efficiency for degradation of specific pro-survival and pro-apoptotic regulators, most notably Bcl-2 interacting killer (BIK) [20,21]. This differential substrate affinity creates a temporal window of proteostatic instability. As β5 levels decline before β5i is fully integrated into active 20S cores, the cell’s capacity to regulate BIK turnover is diminished. IAV exploits this transition through its nucleoprotein (NP), which physically associates with the constitutive β5 subunit [20]. By inhibiting β5 catalytic activity and altering substrate turnover, NP prevents the normal degradative clearance of the pro-survival/pro-apoptotic regulator BIK. The resulting accumulation of BIK disrupts inhibitory Bcl-2/NP complexes, releasing free NP to facilitate viral ribonucleoprotein (vRNP) assembly and viral transcription [20].

### 1.3. The proteasomal switch: kinetics and viral subversion

In the context of IAV, the UPS functions as a dynamic battleground where the host-driven defense mechanisms and viral subversion strategies continuously intersect. While the host uses iP to process viral components for antigen presentation and mitigate oxidative stress, IAV has evolved multi-layered mechanisms to hijack host E3 ligases and deubiquitinating enzymes (DUBs) to facilitate replication [9,22–24].

#### 1.3.1. Host detection and the proteasomal switch kinetics

Early in infection, pattern recognition receptors (PRRs), such as retinoic acid inducible gene-I (RIG-I) and TLR7 detect viral RNA, initiating a signaling cascade through the crucial adaptors mitochondrial antiviral-signaling protein (MAVS) and myeloid differentiation primary response 88 (MyD88), respectively [6]. This signaling axis triggers a surge in the production and secretion of type I and III IFN, which drive the *de novo* transcription of the proteasome 20S subunit beta (PSMB9, PSMB10, and PSMB8) genes, encoding the inducible iP catalytic subunits β1i, β2i, and β5i, respectively, establishing the canonical proteasomal switch (Figure 1A). In standard respiratory models, functional iP complexes become detectable by day two, aligning with peak innate immune pathways to shape the host’s initial antiviral response [9,25,26].

**Figure 1.**
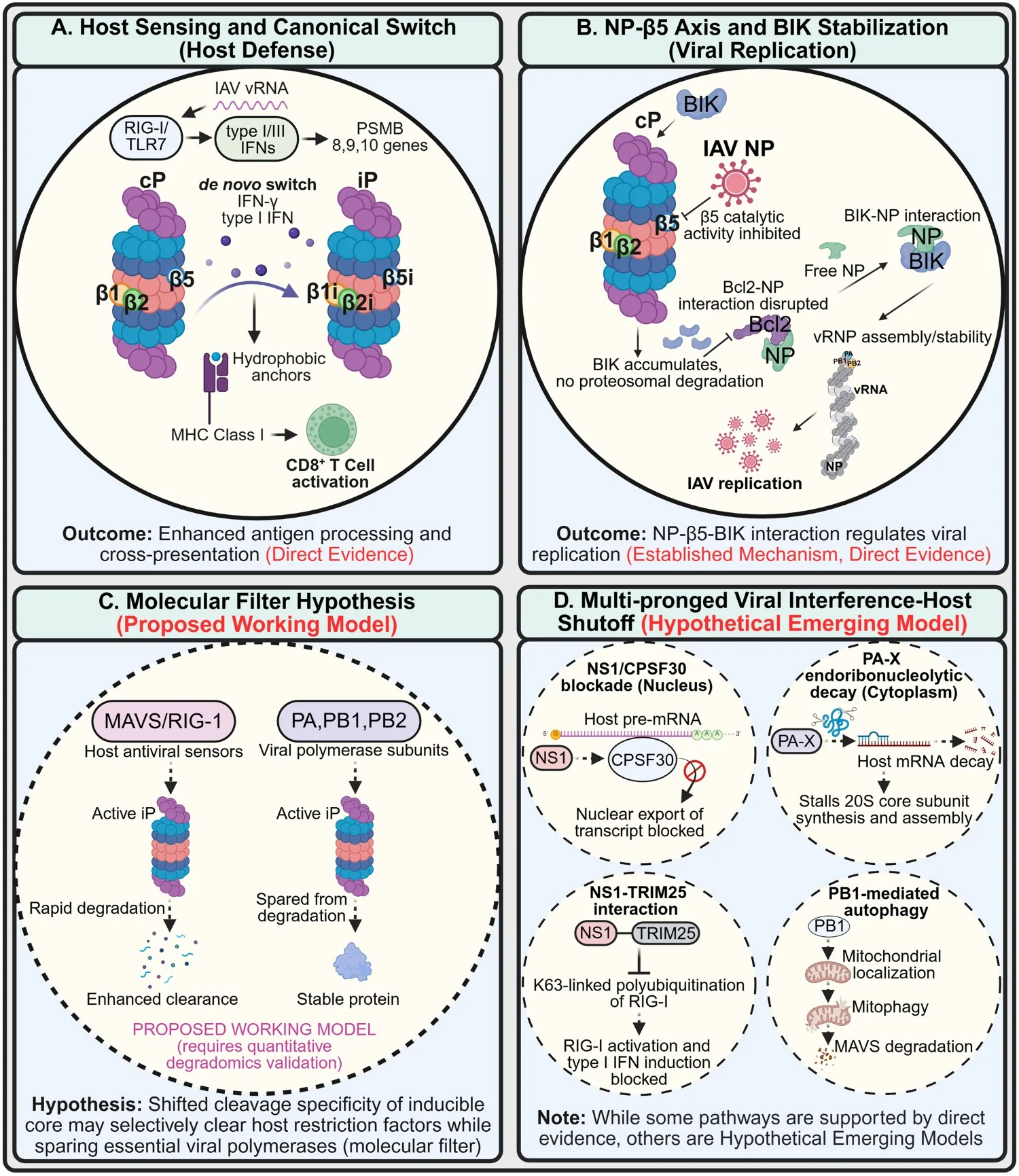
Multi-layered IAV Subversion of Immunoproteasome Biogenesis and Assembly. (A) Host Sensing and Canonical Switch: Detection of viral RNA *via* RIG-I and TLR7 drives type I/III IFN secretion, inducing *de novo* transcription of *PSMB9* (β1i), *PSMB10* (β2i), and *PSMB8* (β5i) to assemble active 20S immunoproteasome (iP) cores that optimize MHC class I antigen presentation. (B) NP-β5 Axis and BIK Stabilization (Established Mechanism): Experimentally verified pathway showing that viral nucleoprotein (NP) selectively binds the constitutive β5 subunit, inhibiting its catalytic cleavage of the pro-apoptotic sentinel BIK. Accumulated BIK disrupts inhibitory Bcl-2/NP complexes, releasing free NP to facilitate vRNP assembly and viral genome replication (solid arrows). (C) Molecular Filter Hypothesis (Unvalidated Working Model): A distinct, speculative working hypothesis proposing that shifting 20S core substrate specificities accelerates the turnover of host antiviral signaling adaptors (such as MAVS and RIG-I) while viral polymerases (PB1, PB2, PA) remain structurally stable. This hypothesis remains unverified and confirmation of this selective filtering requires quantitative degradomics in iP-knockout models. It is visually distinguished from established mechanisms by dashed lines, dashed arrows. (D) Multi-pronged Viral Interference (Hypothetical Emerging Model): IAV deploys coordinated effectors to halt iP biogenesis: nuclear NS1 binds CPSF30 to block host pre-mRNA polyadenylation and export; cytoplasmic PA-X endoribonuclease degrades host transcripts which stalls 20S core subunit synthesis and assembly; NS1 binds TRIM25 to prevent K63-linked polyubiquitination of RIG-I leading to the blocking of RIG-I activation and type I IFN induction; and PB1 targets mitochondria to induce MAVS degradation. Together, these pathways stall *de novo* 20S core biogenesis, establishing a hypothesized kinetic delay in host defense. Definitive evidence demonstrating defective 20S core particle maturation in primary human AECs during early-stage infection, alongside *in vivo* tracking of cell-specific assembly rates are critical knowledge gaps requiring empirical validation. In this figure, solid lines and arrows depict direct, experimentally validated pathways while dashed lines and arrows depict proposed working models. The figure was made using BioRender.

#### 1.3.2. Viral subversion: establishing the kinetic lag

To counter this rapid switch, IAV deploys a coordinated host shutoff program to freeze the proteasome in its baseline constitutive state delaying cP-to-iP transition. However, the magnitude of this suppression is highly strain dependent [27,28]. For instance, strains encoding cellular cleavage and polyadenylation specificity factor 30 (CPSF30)-binding viral non-structural protein 1 (NS1) proteins (such as specific H5N1 isolates and A/WSN/33) or full-length 252-amino-acid polymerase acidic X protein (PA-X) variants (such as H5N1 and pandemic 1918 H1N1) exert robust transcriptional arrest and suppress upstream IFN signaling (Figure 1D). In contrast, strains with attenuated CPSF30 binding (like PR/8 or 2009 pdmH1N1) or truncated 232-amino-acid PA-X proteins exhibit milder mRNA degradation, resulting in a more modest kinetic lag [29,30] (Table 1). Consequently, while the concept of a kinetic lag applies broadly, its duration and biological impact vary significantly across seasonal and high-consequence IAV strains [31,32].

**Table 1.** Summary of influenza A virus (IAV) proteins interacting with the ubiquitin-proteasome system.

Viral protein	Primary target/interactor	Molecular mechanism of action	Biological impact on infection/immunity	References
Matrix Protein 2 (M2)	Autophagy & UPS machinery	Blocks autophagosome-lysosome fusion and interacts with host E3 ligases	Disrupts organellar proteostasis and diverts ubiquitin pools to promote viral budding	[92]
Nucleoprotein (NP)	β5 catalytic subunit (cP)	Binds β5 to inhibit turnover of the pro-apoptotic sentinel BIK	Stabilizes BIK, disrupting Bcl-2/NP complexes to free NP for vRNP assembly and replication	[20,37]
Non-Structural Protein 1 (NS1)	CPSF30 & TRIM25	Inhibits pre-mRNA processing/export; blocks TRIM25-mediated K63-ubiquitination of RIG-I	Induces host shutoff to delay PSMB gene induction; halts type I IFN production	[31,36,79,80,90]
PA-X Endoribonuclease	Host Polymerase II mRNAs	Selective endoribonucleolytic degradation of host mRNA transcripts	Stalls *de novo* synthesis of β1i, β2i, and β5i inducible subunits, causing a kinetic lag	[29,31,32]
PB1	MAVS & Mitochondria	Localizes to outer mitochondrial membrane; targets MAVS for NBR1-mediated selective autophagic degradation	Suppresses MAVS-dependent IFN-β induction, blunting downstream immunoproteasome biogenesis	[38]

Because *de novo* transcription, translation, and proteasome maturation protein (POMP or Ump1)-mediated assembly of active 20S iP require approximately 24–48 h following initial IFN sensing under standard conditions [25,33], early IAV host shutoff effectors (NS1 pre-mRNA processing blockade and PA-X endoribonucleolytic transcript cleavage) are thought to exploit this temporal requirement [31]. We refer to this framework as the proposed kinetic lag model, a putative 24–48 h window of relative proteasomal vulnerability. In this emerging model, we hypothesize that primary viral replication temporarily outpaces inducible host antigen processing and degradative surveillance by delaying functional cP-to-iP core assembly [22,34]. However, we emphasize that direct evidence for impaired functional iP assembly during early IAV infection, particularly in primary human airway epithelial cells (AECs), remains limited. While NS1/PA-X-mediated transcript shutoff is established *in vitro*, direct quantification of active 20S core particle assembly and subunit maturation kinetics in primary respiratory epithelium represents an essential unresolved question.

#### 1.3.3. Subunit sequestration and the molecular filter hypothesis

Beyond global transcriptional arrest, IAV engages in targeted, protein-level manipulation during the proteasomal switch [31,35,36]. Here, it is critical to distinguish between experimentally validated protein interactions and broader theoretical models:

a) The Experimentally Validated NP-β5-BIK Axis: As introduced in Section 1.2, direct biochemical evidence demonstrates that viral NP selectively binds the constitutive β5 subunit, reducing its cleavage of BIK and causing BIK accumulation. This disrupts inhibitory Bcl-2/NP complexes, releasing NP to support vRNP assembly and viral replication [20,37] (Figure 1B) (Table 1). This specific pathway is experimentally grounded by co-immunoprecipitation, pulldown, and functional mutagenesis assays.
b) The Theoretical Molecular Filter Working Model: Extrapolating beyond this verified NP-β5 interaction, we propose a distinct and unvalidated conceptual framework termed the molecular filter hypothesis (Figure 1C). While direct immunopeptidomic and protein half-life profiling during IAV infection remains to be fully established, this model posits a mechanism of viral subversion wherein IAV exploits the altered substrate specificity of iP. In this speculative model, we hypothesize that the transition from cP to iP acts as a biochemical filter that accelerates the degradation of host antiviral signaling molecules (such as MAVS or RIG-I) while selectively preserving viral replication proteins, such as NP and polymerases, which may be recalcitrant to iP-mediated proteolysis [35]. We explicitly emphasize that the molecular filter remains an unverified hypothesis unlike the verified NP-β5 axis. Figure 1C details the molecular filter hypothesis, depicting how viral NS1 and the endoribonuclease PA-X are hypothesized to stall iP assembly, creating a putative kinetic lag in host antiviral signaling and antigen presentation. This multi-layered strategy allows the virus to maintain structural proteins at the levels required for replication while effectively silencing the cell’s ability to alert the adaptive immune system. While global quantitative proteomics data confirm that viral infection markedly reshapes protein turnover rates [22], innate adaptors like RIG-I/MAVS are known targets of proteasomal degradation [36,38], yet the direct iP-dependent degradomics profiling comparing host restriction factor turnover to viral polymerase half-lives has not yet been performed. Consequently, this model serves as a conceptual framework to guide future high-resolution degradomics and quantitative mass-spectrometry studies (Table 2).

**Table 2.** Classification and strength of evidence for key mechanistic concepts in IAV proteasome biology.

Mechanism or framework	Proposed role	Supporting evidence level	Key supporting data and context	Major evidence gaps/validation needed
Host shutoff of iP (NS1/PA-X)	Blocks *de novo* transcription and translation of inducible subunits during early infection	Established/Direct Experimental Evidence	Confirmed CPSF30 binding by NS1 and host mRNA degradation by PA-X in IAV infection [23,31,32,52,80]	Cell-type-specific quantification of 20S core particle maturation rates in primary human airway cells
NP-β5 interaction and BIK stabilization	Suppresses β5 activity to prevent BIK degradation and promote viral replication	Established/Direct Experimental Evidence	*In vitro* co-immunoprecipitation, pulldown, and functional BIK accumulation assays in IAV infection models [20,37]	High-resolution structural mapping of exact NP-β5 contact residues
Putative kinetic lag model	Creates a temporary 24-48 h window of proteostatic vulnerability before full iP assembly	Hypothetical/Emerging Model	Demonstrated delay in cytokine-induced iP assembly relative to early viral replication kinetics *in vitro*; documented NS1/PA-X host shutoff [31,32,59]	Direct evidence for impaired functional 20S core assembly in primary human AECs during early IAV infection; cell-type-specific assembly rates *in vivo*
Intermediate proteasome subversion	Hybrid proteasomes generate suboptimal epitopes, facilitating immune escape	Emerging/Indirect Model	Established existence and unique cleavage kinetics of intermediate proteasomes in non-viral contexts [8,40–42,58,59]	Direct immunopeptidomic identification of intermediate proteasome-derived IAV epitopes
Molecular filter hypothesis	Preferential degradation of host sensors while preserving viral polymerases	Hypothetical/Unvalidated Working Model	Inferred from general proteasome cleavage preferences and viral protein stability	Quantitative degradation profiling and degradomics of viral vs. host proteins in iP-knockout models
Chaperone competition model or ERAD substrate saturation framework	High HA/NA synthesis induces ER stress and translocation of misfolded substrates, temporarily swamping cytosolic 20S core capacity	Hypothetical/Unvalidated Working Model	Inferred from severe ER chaperone demand during viral glycoprotein synthesis	Direct measurement of POMP availability and 20S core assembly during ER stress induction
iP-specific proteostatic buffering in IAV	Inducible 20S cores uniquely prevent UPR-induced epithelial apoptosis by clearing oxidized proteins and DRiPs	Hypothetical/Unvalidated Working Model	Proven resilience of inducible 20S cores under oxidative stress in non-viral/sterile models. General role of UPS in DRiP clearance	Distinguishing iP-specific protection from baseline 26S proteasome, ERAD, and autophagic clearance in IAV-infected primary airway epithelial cells
Persistent iP-driven macrophage inflammation and post-viral fibrosis	Unresolved β5i activity drives chronic macrophage activation, cytokine storms, and TGF-β1-mediated fibrotic remodeling	Extrapolated/Inferred from Non-Viral Models	Well-established in sterile models: bleomycin-induced pulmonary fibrosis [107,109], allergen-induced asthma [110], and LPS-induced lung injury [105,106]	Direct validation in lethal IAV challenge models; distinguishing IAV-specific macrophage kinetics from sterile tissue injury
Biphasic therapeutic modulation (Early activation vs. Late inhibition)	Early activation bolsters antigen presentation and proteostasis; late inhibition limits hyper-inflammatory lung injury and ARDS	Hypothesis-Generating/Partially Validated	Late β5i inhibition is validated in preclinical rodent models of severe lung injury and acute viral infection (MHV-1) [26,107,125,126,134]; early activation is purely conceptual	Early-stage activation lacks validated *in vivo* activators and carries unstudied risks of immunodominant epitope destruction; no clinical trial data in viral ARDS

## 2. The biochemistry of the switch: assembly and regulation

### 2.1. Hierarchical assembly and subunit substitution

The proteasomal switch requires the formation of new 20S particles beginning with a seven-membered α-ring scaffold that sequentially recruits β-subunits. During IAV infection, the rapid induction of β1i and β2i initiates a cooperative assembly pathway; β1i incorporates into the α-ring scaffold more efficiently than the constitutive β1, creating a structural environment that thermodynamically favors the subsequent recruitment of β2i over its constitutive counterpart. This preferential incorporation is driven by the unique binding affinities of the inducible subunits for the nascent β-ring, allowing them to effectively outcompete constitutive subunits for a position in the core [18,39]. The rate-limiting step remains the recruitment of β5i, which features a specialized propeptide that serves as a molecular checkpoint for β-ring maturation and the docking of half-proteasomes. The presence of β1i and β2i significantly accelerates β5i incorporation, ensuring that during the peak of an IFN surge, the biogenesis of full iP is kinetically favored over intermediate proteasome subtypes [40–42].

### 2.2. Chaperone-mediated maturation

The maturation of active 20S core particles requires specialized proteasome-specific chaperones (primarily POMP) [33,43], which coordinate the sequential recruitment of inducible β subunits and facilitates half-proteasome precursor docking at the endoplasmic reticulum (ER) membrane [25,33]. Once the 20S barrel is closed, the pro-peptides of the β subunits undergo autocatalytic cleavage, exposing the N-terminal threonine residues required for proteolytic activity [43]. While established literature focuses on the structure of the completed iP, we propose that the assembly kinetics represent a primary vulnerability in the host’s antiviral response.

We identify two critical synthesis gaps that IAV exploits: (1) The β5i Vulnerability: Because β5i is the mandatory gatekeeper for iP assembly, any viral interference with its translation or folding acts as a biochemical choke point [18,39]. It is hypothesized that IAV-induced host shutoff disproportionately affects iP switch because the system cannot skip the β5i step to form functional alternative complexes. (2) Chaperone Sequestration: We propose a novel theoretical model of chaperone competition, wherein rather than direct physical interaction with POMP, the primary bottleneck during viral glycoprotein synthesis involves ER-associated degradation (ERAD) substrate overload [43–45]. High-level translation and folding of membrane glycoproteins (hemagglutinin [HA] and neuraminidase [NA]) in the ER lumen engage chaperones such as BiP, calnexin, and calreticulin [45]. Accumulation of misfolded glycoprotein species triggers the unfolded protein response (UPR) and drives translocation of ubiquitinated ERAD substrates into the cytosol. This massive surge of misfolded cytosolic substrates competes for general 26S/20S proteolytic core engagement, creating a functional proteostatic bottleneck during early infection without directly depleting cytosolic POMP availability [33,44].

Crucially, this early proteostatic burden is handled broadly by the UPS, ERAD, and autophagic clearance routes; attributing these buffering dynamics specifically to inducible iP cores rather than general proteostasis machinery remains an open question. This framework provides a working model suggesting that the proteasomal switch occurs within a broader, resource-limited proteostatic network that IAV perturbs to favor viral replication [33,45].

### 2.3. Catalytic specificity: shifting the proteolytic landscape

The functional hallmark of the proteasomal switch during IAV infection is a fundamental reconfiguration of the cellular degradative environment. While its architecture remains constant, the replacement of β1, β2, and β5 with their inducible counterparts (β1i, β2i, β5i) redefines the proteolytic core [15]. This transition produces a specialized cleavage profile characterized by increased chymotrypsin-like and trypsin-like activities and a marked reduction in caspase-like activity [46]. The most critical alteration occurs at the β1i site. In cP, the β1 subunit exhibits caspase-like activity, cleaving after acidic residues. However, due to a structural shift in the β1i S1 pocket during the switch to iP, β1i redirects specificity away from acidic residues toward small hydrophobic residues, augmenting total chymotrypsin-like capacity alongside β5i [47]. The iP transition fundamentally alters the proteolytic output of the cell to favor the generation of peptides with hydrophobic C-termini which serve as high-affinity anchors to stabilize the peptide-binding groove of MHC class I molecules and enhance translocation *via* the transporter associated with antigen processing (TAP) [48]. This dual specificity ensures that the viral segments generated during infection are both successfully transported and robustly presented on the cell surface to alert CD8^+^ T cells [10,11,49]. Furthermore, β5i enhances the rate of cleavage after large hydrophobic residues compared to its constitutive counterpart, β5. Interestingly, kinetic studies suggest that the primary advantage of the switch is the quality and specificity of the peptide products rather than simple degradation speed [50].

During IAV pathogenesis, the proteasomal switch has opposing effects on infection. For the host, iP enhances viral antigen processing and promotes viral protein degradation, limiting replication. Conversely, IAV may exploit altered iP cleavage preferences to relieve proteotoxic stress, suppress UPR and apoptosis, and prolong host-cell survival for replication [51]. Although IFN signaling induces iP subunits, IAV counteracts this transition through host-shutoff effectors such as NS1 and PA-X [32,36,44,52]. By suppressing iP assembly and preserving constitutive proteolysis, IAV may create a transient proteostatic window that favors viral protein stability and replication, making the proteasomal switch a potential determinant of disease severity (detailed in Section 4.1).

### 2.4. The double-edged sword of cleavage

Replacing constitutive β subunits with inducible counterparts (β1i, β2i, β5i) shifts proteasomal activity from caspase-like toward enhanced chymotrypsin-like and trypsin-like cleavage. We propose that this catalytic reprogramming reshapes the host–pathogen interface through a testable working model termed the proteolytic timing hypothesis. We hypothesize that the altered proteolytic specificity of iP [46], serves two distinct, non-exclusive functions:

1. Host-Mediated Immunosurveillance: The catalytic shift toward chymotrypsin-like activity optimizes the generation of peptides with hydrophobic C-termini. These segments serve as high-affinity antigenic substrates for MHC class I molecules, effectively turning the viral replication machinery into a signal for CD8^+^ T cell activation [53].
2. Viral Exploitation of Proteostasis: IAV relies heavily on host degradative systems, including constitutive proteasomes, ERAD, and macroautophagy, to manage proteotoxic stress. By rapidly degrading misfolded or spent viral proteins, iP may buffer UPR activation and delay premature apoptosis, thereby prolonging host-cell survival to support viral replication [13,44].

Within the proposed proteolytic timing framework, the catalytic switch presents a functional duality, while generated immunodominant peptides alert host CD8^+^ T cells (Section 3.1), accelerated turnover may simultaneously assist the virus in clearing toxic misfolded defective ribosomal products (DRiPs) to alleviate UPR stress [44,54]. While clearance of DRiPs is a recognized function of the broader UPS, whether inducible iP possesses a specialized, non-redundant capacity over cP to buffer UPR activation and prolong host-cell survival during IAV infection remains hypothetical. The concept that IAV specifically co-opts iP-dependent turnover to delay epithelial apoptosis represents a working hypothesis that must be distinguished from baseline UPS and autophagic quality control. The balance between host immunosurveillance and viral proteostatic benefit is postulated to depend on the duration of this putative kinetic lag [55] (Section 4.3). While enhanced chymotrypsin-like cleavage for MHC class I loading and DRiP clearance during viral stress are individually proven mechanisms, whether IAV relies on iP-mediated proteostatic buffering to delay host cell apoptosis remains a working hypothesis requiring further validation in subunit-knockout models (Table 2).

### 2.5. Non-canonical immunoproteasome functions in antiviral defense

While iP was classically identified for its role in generating MHC class I peptides, emerging evidence highlights its essential non-canonical functions in maintaining cellular homeostasis during respiratory viral infections. IAV infection induces a severe wave of reactive oxygen species (ROS) in infected bronchial epithelial cells. Oxidative damage causes widespread protein unfolding and carbonylation. Studies in sterile non-viral models demonstrate that, unlike cP, inducible 20S cores exhibit resistance to oxidative inactivation and maintain proteolytic efficiency toward poly-oxidized, misfolded protein aggregates *in vitro*. In infected airway tissues, oxidative proteotoxicity is managed through cooperative interplay among cP, inducible iP, ERAD, and selective macroautophagy/mitophagy [42,56]. This protects the respiratory epithelium from oxidative proteotoxic collapse and premature cell death. Beyond IκBα degradation in the NF-κB pathway, iP regulates the duration and magnitude of type I and III IFN responses. By controlling the turnover kinetics of key innate signaling adaptors, including MAVS, RIG-I, and activated STAT1 dimers, iP prevents excessive or prolonged ISGs expression, helping to restore baseline homeostasis following peak infection [7]. During acute IAV infection, mitochondrial dysfunction triggers the accumulation of damaged mitochondrial outer membrane proteins. iP operates in tandem with macroautophagy and mitophagy (Parkin/PINK1-mediated clearance). When high-volume proteasomal substrate loads threaten to saturate 20S core capacity, ubiquitin-tagged complexes are redirected to p62/SQSTM1-dependent selective autophagy [38,57]. Conversely, iP activity clears outer mitochondrial membrane proteins to facilitate the engulfment of damaged mitochondria by autophagosomes, illustrating vital crosstalk between proteasomal and autophagic clearance routes during viral stress. However, direct evidence demonstrating that this biochemical resilience confers an iP-specific protective shield in the respiratory epithelium during IAV infection is currently lacking. Identifying the precise contribution of iP catalytic subunits relative to these alternative degradative systems requires rigorous evaluation in subunit-specific knockout systems.

### 2.6. Intermediate (Hybrid) proteasomes: structural assembly and functional divergence

While the proteasomal switch is frequently framed as a binary transition from cP to iP, significant populations of intermediate (hybrid) proteasomes coexist within infected and cytokine-stimulated tissues [8,58]. Understanding the distinct assembly pathways, catalytic properties, and biological significance of these hybrid forms is essential for a complete model of proteasome biology during viral infection. Intermediate proteasomes are asymmetric or symmetric 20S core particles that incorporate a mixture of constitutive and inducible catalytic subunits [58]. The two predominant forms are β5i-single intermediate (β1-β2-β5i), which is formed when β5i is incorporated alongside constitutive β1 and β2 subunits, and β1i/β5i-double intermediate (β1i-β2-β5i), formed when both β1i and β5i are recruited, retaining only the constitutive β2 subunit [58].

In uninfected non-immune cells, intermediate proteasomes account for up to 10–20% of the total proteasome pool, but this proportion surges to 30–50% during the initial 12–36 h of IFN-γ exposure or viral sensing, representing a dominant transitional state before full iP assembly is achieved [58,59]. Intermediate proteasomes are not merely incomplete assembly artifacts; they possess distinct, non-redundant enzymatic properties [8,58,59]. β5i-single intermediate uniquely couples the enhanced chymotrypsin-like cleavage of β5i with the robust caspase-like activity of constitutive β1 [8,58]. This hybrid catalytic profile allows intermediate proteasomes to process a unique subset of antigenic peptides that neither cP nor full iP can efficiently generate [58]. Quantitative immunopeptidomics reveals that specific tumor- and viral-derived epitopes are exclusively liberated by intermediate proteasomes [8,58]. During IAV infection, these hybrid complexes act as an essential biochemical bridge; they expand the breadth of the MHC class I ligandome during early infection, ensuring antigenic presentation even before fully mature iP barrels dominate the cellular degradative landscape [8,58]. These hybrids are catalytically active and possess unique, mosaic cleavage specificities that differ from both pure cP and full iP complexes, they represent a less specialized transitional state that is hypothesized to contribute to the proposed kinetic lag by being less efficient at generating immunodominant epitopes compared to fully mature iP complexes.

### 2.7. Non-Technical Summary: Simple Explanation of the Proteasome Switch

Constitutive proteasome (cP) is the cell’s everyday recycling plant that breaks down worn-out proteins into basic amino-acid building blocks. When a cell detects a viral infection like influenza, inflammatory signals prompt it to upgrade these recycling plants into specialized security centers called immunoproteasomes (iP). These upgraded units now chop up viral proteins into specific, high-affinity peptide tags that are displayed on the cell surface. This surface display acts as a biochemical red flag that alerts passing immune cells (CD8^+^ T cells) to destroy the infected cell before the virus can replicate and spread.

## 3. The classical role: host immunity *via* antigen presentation

### 3.1. Epitope generation: the molecular filter of viral defective ribosomal products (DRiPs)

The host’s survival during IAV infection depends on the rapid reconfiguration of the cell’s antigenic identity to facilitate immune recognition. This process relies on the generation of 8- to 10-amino-acid peptides from internal viral proteins, primarily matrix 1 (M1), NP, and polymerase basic protein 1 (PB1), which are loaded onto MHC-I molecules for CD8^+^ T cell recognition [60,61] (Table 1). The IFN-induced switch to iP is a host adaptation to the chemical composition of these viral proteins. Dominant epitopes, such as M1_58-66_ and NP_366-374_, are enriched with large hydrophobic residues [62,63]. iP’s specialized S1 pocket preferentially cleaves after these residues, ensuring the efficient liberation of peptides with the precise C-terminal anchors required for high-affinity binding to TAP and MHC-I [19,47,64].

Timing is critical, as the host must initiate the presentation of these antigens during the initial wave of replication to establish the signaling required for eventual clearance. This is achieved by degrading DRiPs, misfolded or prematurely terminated polypeptides produced at high rates during viral translation [65]. The iP switch enhances the cell’s ability to filter this proteomic noise into high-affinity segments. During high-speed viral replication, a significant portion of viral polypeptides are DRiPs, aggregate-prone and potentially toxic to the cell [48,66]. While cP may be overwhelmed by these species, iP’s increased chymotrypsin-like activity allows for the rapid conversion of these viral products into immune signals. Beyond the rapid turnover of DRiPs and the β5-BIK axis, iP coordinates broader aspects of antigen processing. It enhances cross-presentation efficiency in professional antigen-presenting cells (APCs), particularly CD8α^+^ dendritic cells (DCs), by generating longer precursor peptides that undergo precise trimming by aminopeptidases in the ER [48,64,67]. Furthermore, the altered cleavage preferences of iP subunits facilitate the generation of peptides from highly stable, long-lived viral structural proteins (such as M1 and NP) that are otherwise recalcitrant to cP degradation. This dual capability, processing both short-lived DRiPs and stable structural assemblies, ensures a comprehensive antigenic representation across all stages of the viral replication cycle.

In iP-deficient models, the reduction of immunodominant epitopes grants the virus a kinetic advantage, leading to uncontrolled replication and severe lung pathology [10,11,68,69]. We posit that iP functions as a proteomic noise filter, converting viral waste into the primary substrates for immunosurveillance. Although the systemic recruitment of cytotoxic T lymphocytes (CTLs) to the lungs typically peaks after the initial viral surge, the early generation of these epitopes is a prerequisite for the maturation and activation of DCs [70]. By filtering DRiPs into antigens within the first 24–48 h, iP ensures that DCs can effectively prime the adaptive immune system, determining the trajectory toward viral resolution rather than lethal pathogenesis. Evaluating iP’s role in processing M1, NP, and PB1 makes it clear that the proteasomal switch is not merely a homeostatic adjustment, but a primary determinant of host immunosurveillance and viral clearance.

### 3.2. CD8^+^ T cell priming and the signaling threshold

iP is a primary requirement for the initiation of the adaptive immune response. In DCs, iP facilitates the cross-presentation of viral antigens, which is necessary for the activation and expansion of virus-specific CTLs [71]. Research identifies a T-cell intrinsic defect in iP-deficient models; for instance, LMP2^-/-^ and MECL-1^-/-^ mice exhibit a selective reduction in CD8^+^ T cell populations, a deficit that persists even when cells develop in a normal thymic environment [69,72]. Beyond its role in peptide generation, iP functions as a signaling regulator by modulating the NF-κB pathway. The transition to iP enhances the degradation of IκBα, the inhibitory protein that sequesters NF-κB in the cytoplasm. By accelerating the turnover of IκBα and facilitating the processing of the p105 precursor into its active p50 form, iP promotes the nuclear translocation of NF-κB [73–75]. This increased signaling throughput drives the transcription of essential pro-inflammatory cytokines, including IFN-β and TNF, which are necessary to shape the magnitude of the cellular immune system [11,76]. Consequently, iP is required for efficient antigen presentation and the establishment of a robust signaling threshold during the early stages of IAV infection.

### 3.3. Beyond antigen presentation: the subversion-as-regulation model

IAV-mediated subversion of the proteasome involves the redistribution of host degradative capacity to favor viral protein stability over host restriction factors [77,78]. The virus utilizes host E3 ubiquitin ligases to redirect proteolysis towards critical immune signaling hubs, as seen in the NS1-mediated interference with host ubiquitin cascades [79–81]. A primary example of this redirection is the viral factor-induced degradation of TAP, which prevents the translocation of peptide cargo into the ER and subsequently reduces the density of MHC-I complexes on the cell surface [82,83]. This subversion is characterized by the concurrent protection of the viral proteome and the stabilization of host pro-apoptotic factors [20,23,84]. IAV recruits host DUBs and viral chaperones to prevent the polyubiquitination of replication machinery, including NP and viral polymerases. Simultaneously, the virus induces the selective suppression of the β5 subunit. By stabilizing pro-apoptotic factors such as BIK, which facilitates viral transcription, the virus creates a period of proteostatic imbalance that benefits replication [20,85].

As detailed in Section 1.2 and 1.3.3, NP selectively targets the constitutive β5 subunit to inhibit BIK turnover. Rather than repeating this molecular mechanism here, we highlight its immunological consequence, by hindering the *de novo* incorporation of β5i into active 20S cores, the virus delays immunodominant epitope presentation, effectively raising the threshold required for early CD8^+^ T cell priming (Figure 1B). Furthermore, the coordinated actions of NS1 and PA-X in driving host shutoff stall the assembly of functional iP, creating a window of permissive replication that may exacerbate pulmonary pathology. The outcome of this competition, determined by whether the host can achieve iP-mediated clearance before the virus-driven dysregulation leads to a failure in homeostatic attenuation and triggers a cytokine storm, ultimately governing the trajectory of pulmonary pathogenesis [86,87] (Figure 2).

**Figure 2.**
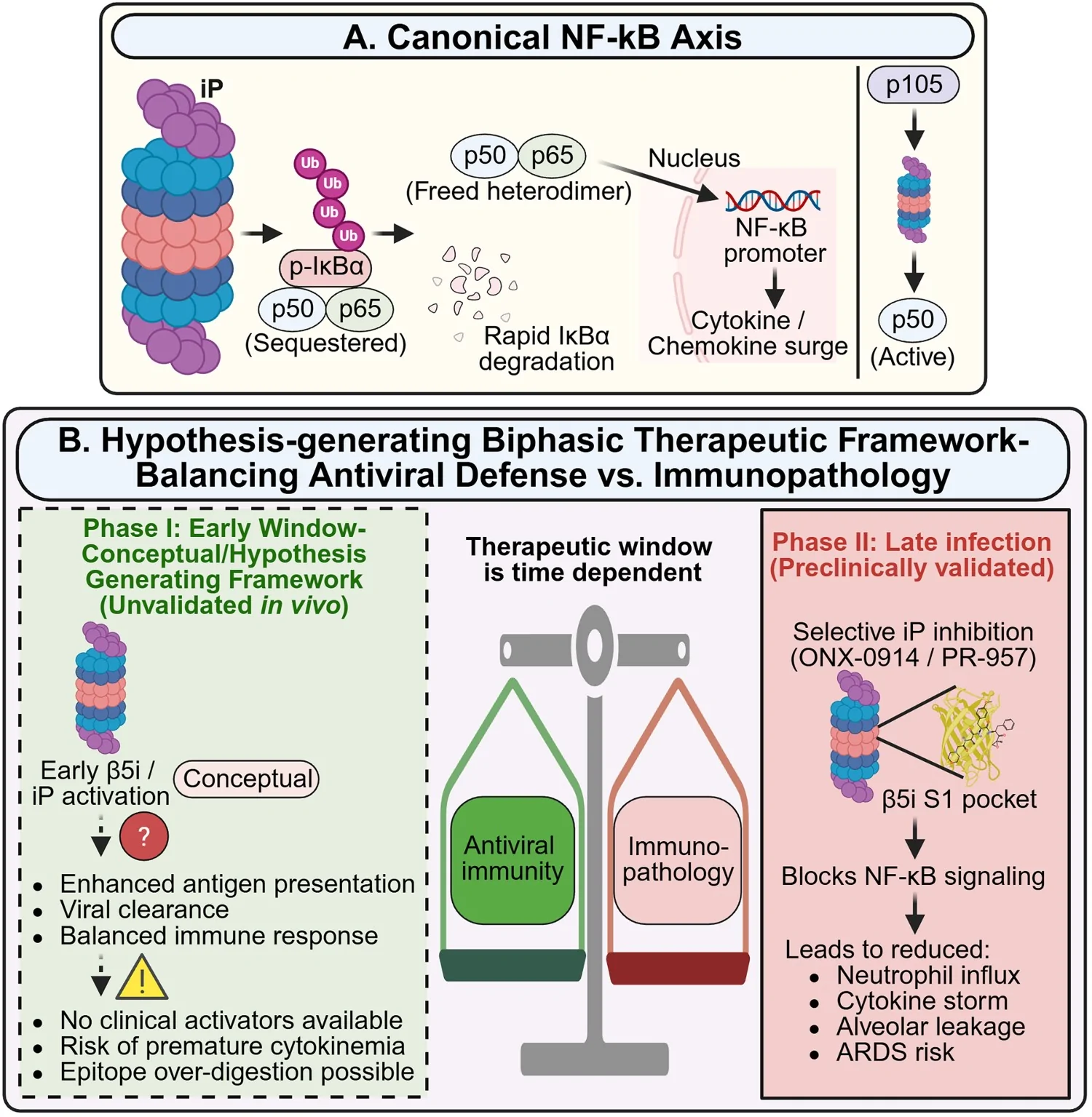
Immunoproteasome Regulation of NF-κB Cascades and Biphasic Therapeutic Windows. (A) Canonical NF-κB Axis: Upon viral challenge, inducible 20S immunoproteasome (iP) cores containing catalytic subunits β1i (LMP2), β2i (MECL-1), and β5i (LMP7) accelerate the targeted degradation of phosphorylated, polyubiquitinated IκBα, releasing sequestered p50/p65 heterodimers. The resulting liberated p50/p65 complexes translocate to the nucleus, binding NF-κB promoter elements to drive the transcription of pro-inflammatory cytokines, chemokines, and vascular adhesion molecules. Concurrently, iP cores mediate the limited proteolytic processing of the p105 precursor into active p50 subunits. (B) Hypothesis-Generating Biphasic Framework-Balancing Antiviral Defense vs. Immunopathology. Conceptual schema outlining a timing-dependent framework for iP modulation. Left-Phase I: Early Window-Conceptual/Hypothesis Generating Framework (Unvalidated *in vivo*): During early viral replication, the host relies on inducible iP biogenesis to conceptually clear defective ribosomal products (DRiPs) and process high-affinity peptides for MHC class I surface presentation to prime CD8^+^ cytotoxic T lymphocytes (CTLs). Pharmacological stimulation of this early phase remains a theoretical, unvalidated concept, as no selective small molecule iP activators are approved for clinical use, and premature modulation carries substantial risks of systemic cytokinemia and immunodominant epitope over-digestion. Conversely, premature inhibition during this window impairs CTLs priming and leads to elevated viral loads. Right-Phase II: Late infection (Preclinically validated): In severe influenza pneumonia, peak viral replication has typically subsided, and disease severity is driven primarily by an overexuberant myeloid cytokine storm and neutrophil recruitment. Administration of selective β5i inhibitors (such as PR-957/ONX-0914), which covalently dock into the specialized chymotrypsin-like S1 substrate-binding pocket of β5i, serves as an evidence-based pharmacological emergency brake. Selective β5i blockade dampens hyper-active NF-κB signaling, attenuating alveolar epithelial desquamation, capillary leakage, and acute respiratory distress syndrome (ARDS) without the dose-limiting toxicities of non-selective pan-proteasome inhibitors. It should be emphasized that the suppression of alternative macrophage polarization and TGF-β1 signaling *via* β5i inhibition has been demonstrated almost exclusively in sterile experimental paradigms; extending these mechanisms to post-viral lung injury following IAV remains a speculative model awaiting direct *in vivo* testing. Solid lines and arrows depict experimentally validated biological pathways; dashed lines and arrows depict theoretical concepts and working models. The figure was made using BioRender.

## 4. Viral subversion: IAV as a proteasomal saboteur

### 4.1. Orchestrating the host shutoff: NS1, PA-X, and PB1

For IAV, the UPS is a critical regulatory node that it must manipulate to facilitate its own replication while neutralizing host antiviral factors [23,88]. The virus employs a coordinated host shutoff program, primarily mediated by NS1 and PA-X, which is hypothesized to stall the functional cP-to-iP switch, establishing a putative kinetic delay [31]. These proteins inhibit the transition from constitutive to inducible subunits, thereby prioritizing the translation of viral mRNAs while blunting the IFN-mediated upregulation of iP [6,31].

NS1, the most versatile effector in the IAV arsenal, rewires host ubiquitination machinery by primarily targeting E3 ubiquitin ligases [36,79,80,89]. In addition to CPSF30 and PA-X host shutoff [31,52], NS1 targets TRIM25 to prevent RIG-I K63-polyubiquitination [36], while PB1 triggers selective MAVS autophagic degradation [38], systematically disabling upstream IFN signaling prior to PSMB gene induction (Figure 1D) [36,80]. Furthermore, NS1 interacts with the 19S regulatory subunit of the proteasome and promotes the degradation of specific host factors, such as sphingosine 1-phosphate lyase, to remove inhibitory proteins that restrict viral fitness [90,91]. Host intrinsic immunity utilizes distinct ubiquitin-dependent degradation pathways to suppress viral assembly and egress. For example, the membrane-associated E3 ubiquitin ligase MARCH8 catalyzes K63-linked polyubiquitination of the IAV M2 envelope protein, targeting M2 for lysosomal degradation to block viral particle release [92] (Table 1).

We propose that NS1 and PA-X do not merely arrest host protein synthesis but strategically reallocate the cell’s degradative capacity. By inducing a global host shutoff, the virus reduces the intracellular pool of competing host transcripts. Our hypothesis is that by stalling the assembly of iP, the virus ensures that the available cP is directed towards viral requirements, such as the regulation of viral polymerase activity, rather than being utilized for the degradation of ISGs and antigen presentation. Consequently, host shutoff serves as a proteostatic requirement for the virus, preventing the proteasome from being saturated by the hundreds of ISGs that would otherwise be induced during the host’s antiviral response.

### 4.2. Nucleoprotein selectivity: The β5 vs. β5i strategic choice

IAV NP is a primary determinant of viral fitness that modulates cellular degradative capacity [93]. Mechanistically, NP selectively interacts with the 20S catalytic core *via* the constitutive β5 subunit, as detailed in Sections 1.2 and 1.3.3 [20] (Figure 1B). By engaging β5 during early infection, NP may hinder the *de novo* recruitment and propeptide processing of incoming β5i subunits, thereby contributing to delayed maturation of fully assembled iP cores. As discussed in Section 2.3, a delayed cP-to-iP switch temporarily preserves constitutive proteolysis. During this transition, β1 replacement by β1i shifts cleavage from acidic residues toward small hydrophobic residues, enhancing chymotrypsin-like activity, while β5i increases cleavage after large hydrophobic residues relative to β5 (Table 3) [47]. Thus, delayed iP assembly may reduce the timely generation of high-affinity C-terminal anchors required for optimal MHC class I antigen presentation [15,46]. Hence, NP may not suppress β5 expression or maintain cP architecture per se but rather modulate proteasome function by inhibiting β5-mediated catalytic turnover of pro-viral regulators while delaying β5i incorporation.

**Table 3.** Biochemical & functional comparison of constitutive proteasome (cP) vs. immunoproteasome (iP) during influenza A virus infection.

Biological feature	Constitutive proteasome (cP)	Immunoproteasome (iP)	Biochemical & functional impact of cP-to-iP transition	References
Catalytic subunits	β1, β2, β5	β1i, β2i, β5i	Alters cleavage specificity (toward hydrophobic C-termini) and peptide repertoire	[47,135]
Induction/Expression pattern	Constitutively active across most nucleated cells	Induced by IFN-γ, type I and III IFN, and oxidative stress	Transforms basal housekeeping proteasome into an inducible, high-efficiency antiviral degradative core	[7,136]
Peptide cleavage profile	Cleaves after acidic, basic, and hydrophobic residues	Hydrophobic C-terminal anchor optimization	Increases binding affinity of generated peptides for TAP translocation and MHC class I loading	[19,97]
Antigen presentation	Generates baseline self-peptides for immune tolerance	Optimizes immunodominant viral epitope production	Enriches MHC-I surface loading with viral DRiP-derived peptides, enhancing CD8^+^ T cell priming	[11,76]
Inflammatory Signaling	Manages basal IκBα turnover	Accelerates IκBα turnover and p105 processing	Accelerates canonical NF-κB nuclear translocation, amplifying pro-inflammatory cytokine surges	[73,75]
Proteostatic Buffering	Susceptible to oxidative inactivation	Highly resistant to ROS-induced inactivation	Rapidly clears oxidized and misfolded viral/host proteins to prevent UPR-induced epithelial apoptosis	[9,10,11,46]

Notably, the IAV structural residues are highly conserved across diverse IAV lineages, including human H1N1, H3N2, and avian/human H5N1 strains, as evidenced by sequence alignment [94]. This indicates that NP-mediated β5 suppression represents a broadly conserved mechanism of proteasomal manipulation, whereas the downstream inflammatory consequences are further amplified in highly pathogenic avian influenza strains due to hyper-induction of host cytokines. While host E3 ligases like TRIM41 target NP for polyubiquitination to limit infection [95], the virus recruits host DUBs (such as USP11) to remove these ubiquitin chains. This enzymatic reversal stabilizes NP during the critical transition from viral transcription to genome replication [96].

### 4.3. Viral proteostatic control during the immunoproteasome switch

The cP-to-iP transition represents a major reorganization of cellular proteolysis that IAV must navigate to sustain infection. Although this host adaptation enhances MHC-I peptide generation [11,23,76,97], IAV may exploit the altered cleavage landscape to promote viral fitness [9,22]. The reconfigured 20S core changes substrate specificity and degradation kinetics [11,46,47,89]. We propose that some viral proteins are intrinsically less susceptible to iP-mediated degradation, while viral NP-mediated suppression of β5 preserves replication machinery for vRNP assembly [20,91]. This interaction may delay β5i incorporation, stall the proteasomal switch, and limit the generation of immunodominant epitopes.

A significant facet of this viral strategy involves the sequestration of proteasomal capacity. During rapid replication, a high volume of DRiPs, misfolded or prematurely terminated polypeptides, are generated. These aggregate-prone species could saturate the catalytic sites of the proteasome, creating a kinetic bottleneck. This saturation may prevent the efficient turnover of primary replication enzymes and stable pro-viral host factors while prioritizing the clearance of host antiviral signaling molecules [25,54]. To sustain replication under host proteotoxic stress, IAV must navigate the structural constraints of the 20S switch. Having established the specific actions of NS1/PA-X (Section 4.1) and NP-β5 (Section 4.2), the primary kinetic outcome is the creation of a temporary window where viral replication machinery remains stable while host pre-mRNA processing is arrested (Figure 1) [20,32,36,98]. If the virus successfully maintains its core proteome (PB1, PB2, and PA) while systematically clearing host mediators (like MAVS or RIG-I), the proteasomal switch facilitates viral subversion rather than host defense [23]. This tactical preservation amidst increased cellular proteolysis allows IAV to achieve high replication titers while simultaneously delaying the signaling required for a robust adaptive immune response. Ultimately, the trajectory of pulmonary pathogenesis is determined by whether the host can establish iP-mediated clearance before the virus-driven dysregulation leads to a failure in homeostatic attenuation [93].

### 4.4. Strain-specific diversity and model-specific context

Mechanistic insights into proteasomal manipulation are highly dependent on viral strain polymorphisms and the experimental host systems used. Strains with high-affinity CPSF30 binding (e.g. H5N1, A/WSN/33) efficiently suppress host pre-mRNA processing, blocking PSMB8/9/10 induction [27]. Strains lacking this binding site rely predominantly on PA-X endonuclease activity [30]. The presence of a full-length 252-aa PA-X protein (containing nuclear localization residues 195 K/198 K/199R) accelerates host transcript degradation compared to truncated 232-aa variants found in select human seasonal strains [28,29]. While continuous cell lines (e.g. HEK293 T) provide tractable biochemical systems, polarized primary human bronchial epithelial cells (cultured at an air–liquid interface) exhibit rapid, tissue-specific iP induction patterns that more accurately reflect *in vivo* human infection dynamics [99,100].

### 4.5. Insights from high-throughput omics: single-cell RNA sequencing, spatial transcriptomics, and immunopeptidomics

Recent advances in systems biology have provided unprecedented resolution into the spatiotemporal dynamics of the proteasomal switch during IAV infection. Single-cell RNA sequencing (scRNA-seq) of infected lung tissues demonstrates that iP induction is not uniform across the respiratory architecture [101]. While recruited interstitial macrophages and CD8α^+^ DCs exhibit rapid, robust upregulation of iP transcripts within 24 h of infection, AECs display variable induction kinetics. Spatial transcriptomics further reveal distinct microenvironmental niches, alveolar regions with high viral load exhibit localized repression of host PSMB transcripts due to NS1/PA-X activity, surrounded by an outer ring of uninfected bystander cells undergoing intense IFN-driven iP biogenesis. Further, high-resolution quantitative proteomics has established that IAV infection induces widespread re-wiring of cellular protein turnover rates. Pulsed stable isotope labeling by amino-acids in cell culture and degradomics profiling confirm that active 20S core subunit composition shifts dynamically, providing physical proof of the delay in functional β5i incorporation during early viral replication [22]. Direct isolation and liquid chromatography-tandem mass spectrometry sequencing of MHC class I-bound peptides (the immunopeptidome) demonstrate that iP assembly alters nearly half of the presented peptide repertoire. In IAV infection, immunopeptidomics confirms that immunodominant epitopes derived from viral M1 and NP depend specifically on iP subunits cleavage preferences for efficient C-terminal anchor generation [102].

## 5. Pathogenesis: immunoproteasome in lung injury and inflammation

### 5.1. Dynamic immunoproteasome kinetics across the IAV pathological cascade

Rather than functioning as a static correlate of tissue damage, iP assembly and activity undergo profound spatiotemporal shifts across the sequential stages of IAV pathogenesis (Figure 3), detailed below. While some of these processes and concepts have been empirically validated in published studies, others remain theoretical propositions and are presented as such.

- Phase 1: Early Infection and Kinetic Delay (0-24 h post-infection (hpi)): At baseline, respiratory AECs maintain low levels of iP subunits, relying predominantly on cP [67]. Initial viral sensing *via* RIG-I and TLR7 triggers local type I/III interferon IFN secretion (details in Section 4.1) [36]. However, early viral replication deploys NS1 and PA-X host shutoff mechanisms to block PSMB pre-mRNA nuclear export and degrade host transcripts [31]. This viral interference is proposed to create a putative kinetic lag, theoretically stalling *de novo* 20S core biogenesis and allowing viral genome replication to outpace the host’s degradative surveillance during early infection [34].
- Phase 2: Epithelial Switch and Proteostatic Shield (24-48 hpi): As autocrine and paracrine IFN-β/γ signaling intensifies, AECs overcome viral host shutoff, driving robust PSMB8/9/10 transcription and POMP-mediated 20S iP assembly [33]. During this peak replication phase, the primary functional role of iP in infected epithelium is proteostatic buffering [9,46,103]. We hypothesize that inducible 20S cores participate alongside the broader UPS, ERAD, and autophagic machinery in proteostatic buffering. While the general UPS rapidly turns over misfolded viral DRiPs and damaged host proteins to delay UPR-mediated apoptosis, direct evidence that this cytoprotective effect is uniquely dependent on immunoproteasome subunits, rather than baseline proteasome or autophagic flux, remains to be established empirically in primary AECs (details in Section 3.1) [44,55]. Concurrently, altered catalytic cleavage preferences generate hydrophobic C-terminal anchor peptides for MHC-I loading, marking infected cells for CD8^+^ CTLs recognition [15,46].
- Phase 3: Myeloid Hyper-Activation and Acute Lung Injury (48-96 hpi): As IAV infection progresses, massive recruitment of monocyte-derived alveolar macrophages (AMs) and neutrophils shifts the dominant pulmonary proteasome pool toward inducible cores [104]. While the direct causal link between iP hyper-activation and acute alveolar injury has been primarily demonstrated in sterile endotoxin and non-viral pulmonary injury models [105,106], in IAV infection infiltrating myeloid subsets display profound induction of iP subunits. By extrapolating from these non-viral paradigms, we hypothesize that iP expression is hyper-induced, accelerating IκBα turnover and p105 processing into active p50 subunits (details in Section 3.2). This hyper-active NF-κB signaling drives an unresolvable cytokine surge, triggering extensive alveolar epithelial desquamation, tight-junction loss, and capillary leak, culminating in viral acute respiratory distress syndrome (ARDS) [9,105].
- Phase 4: Resolution vs. Fibrotic Remodeling (96+ hpi): Following viral clearance, iP expression gradually normalizes in surviving epithelial populations [67]. However, in severe post-viral lung injury, whether persistent iP activation directly drives post-viral fibroproliferation remains an open empirical question. Mechanistic studies establishing that persistent β5i activity promotes TGF-β1-dependent myofibroblast differentiation and aberrant collagen deposition derive largely from sterile models of pulmonary fibrosis (such as bleomycin challenge) [107–110]. Extrapolating these findings suggests that unresolved myeloid and stromal iP signaling may similarly contribute to post-viral fibrotic remodeling following severe IAV pneumonia, representing a critical area for direct experimental validation in viral models [75,111,107,112–115] (Figure 3).

**Figure 3.**
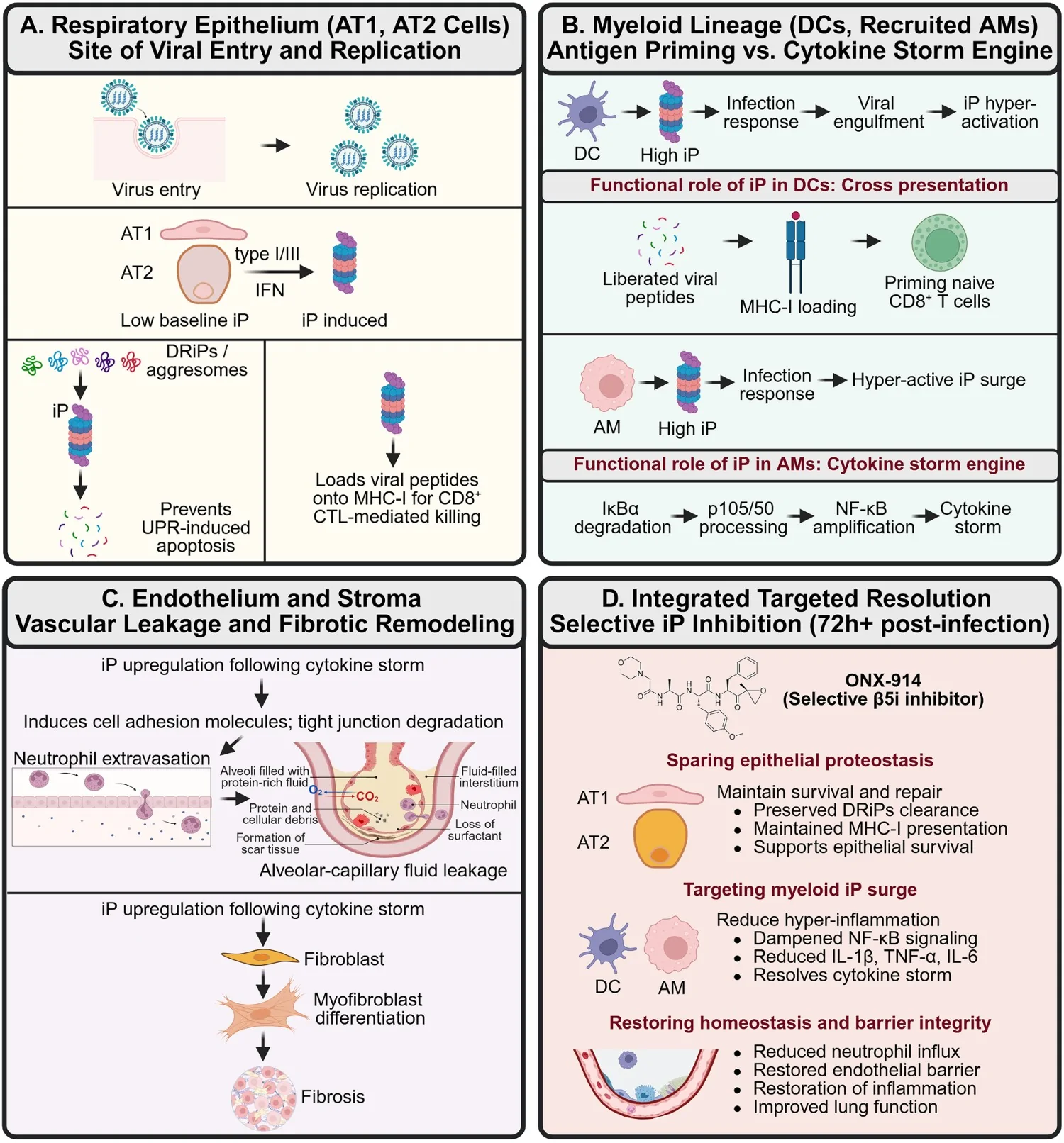
Integrated Landscape of IAV Pathogenesis and Cell-Type Heterogeneity, and Targeted Intervention. Schematic depicting cell-type-specific immunoproteasome (iP) biology across the respiratory tract during influenza A virus (IAV) infection, mapping the progression from viral replication and innate buffering to hyper-inflammation, barrier destruction, and pharmacological resolution. (A) Respiratory Epithelium (AT1 and AT2 Cells-Site of Viral Replication and Proteostatic Buffering): Non-immune alveolar epithelial cells (type I and type II AECs) maintain low baseline iP subunit (β1i, β2i, β5i) expression, which is rapidly induced *de novo* following type I and type III interferon (IFN) secretion. In these cells, inducible 20S iP cores perform two essential functions: (1) Proteostatic Shielding: Rapidly clearing misfolded viral defective ribosomal products (DRiPs) and carbonylated host aggresomes, preventing toxic proteotoxic stress, unfolded protein response (UPR) activation, and premature epithelial cell apoptosis; and (2) Direct Antigen Presentation: Generating viral peptides containing hydrophobic C-terminal anchors (such as M1_58-66_ and NP_366-374_) for high-affinity MHC class I surface loading, marking infected epithelium for targeted lysis by cytotoxic CD8^+^ T lymphocytes (CTLs). (B) Myeloid Lineage (DCs and Recruited Alveolar Macrophages-Antigen Presentation and Cytokine Orchestration): In professional myeloid cells, iP subunits are constitutively expressed at high baseline levels. (1) In CD8^+^ dendritic cells (DCs), high iP activity is essential for viral antigen cross-presentation, generating extended precursor peptides to prime naive CD8^+^ T cells in draining lymph nodes. (2) In contrast, upon massive infiltration of bone-marrow-derived monocyte/macrophages into the alveolar space during peak infection, hyper-active iP cores accelerate IκBα degradation and p105/50 processing, driving an unresolvable NF-κB amplification loop. This hyper-activation triggers an uncontrolled cytokine storm, driving alveolar epithelial desquamation and acute respiratory distress syndrome (ARDS). (C) Endothelium and Stroma (Vascular Permeability and Fibrotic Remodeling): (1) In microvascular endothelial cells, iP upregulation downstream of circulating inflammatory cytokines accelerates the turnover of tight junction complexes and induces the expression of vascular cell adhesion molecules, promoting massive neutrophil extravasation, vascular hyperpermeability, and alveolar proteinaceous fluid flooding. (2) In interstitial stromal fibroblasts, persistent iP activation drives TGF-β1-dependent myofibroblast differentiation and excessive collagen deposition, establishing a direct mechanistic link between unresolved proteasomal activity and post-viral interstitial pulmonary fibrosis. (D) Integrated Targeted Resolution (Selective β5i Inhibition Window): Superimposing the preclinically validated therapeutic window for selective β5i inhibitors (such as ONX-0914 / PR-957) administered during late-stage hyper-inflammation (72h+ post-infection). By selectively docking into the unique S1 substrate-binding pocket of LMP7 (β5i), ONX-0914 dampens excessive myeloid NF-κB signaling and resolves the pro-inflammatory cytokine surge without impairing baseline constitutive proteasome (cP) housekeeping functions in respiratory epithelial cells. This cell-type-specific dampening halts neutrophil influx, restores alveolar-capillary barrier integrity, prevents ARDS-associated mortality, and limits long-term fibrotic remodeling. Mechanistic evidence linking β5i inhibition to suppressed macrophage activation and TGF-β1 fibrotic signaling stems primarily from sterile models, providing a conceptual framework that requires direct confirmation in severe IAV infection. The figure was made using BioRender.

### 5.2. ARDS, alveolar integrity, and fibrosis

In the healthy lung, iP expression remains at basal levels but is rapidly inducible by IFN to facilitate viral antigen presentation [67]. While early iP induction is generally protective, as evidenced by increased viral titers and mortality in iP-deficient models, sustained or dysregulated activity contributes to pulmonary pathology [10,11,68,69]. Severe IAV infection often results in extensive alveolar epithelial desquamation and ARDS [104,105]. Within the alveoli, type I and II AECs undergo both lytic and programmed cell death, disrupting the tight junctions necessary for maintenance of the alveolar-capillary barrier. This loss of barrier integrity permits the leakage of proteinaceous fluid into the alveolar space, a hallmark of ARDS [104,105]. While iP acts as an essential proteostatic buffer during early viral replication [9–11], persistent iP hyper-activation in recruited monocyte-derived macrophages fuels an unresolvable NF-κB cytokine storm (TNF, IL-1β, IL-6) that exacerbates tissue injury [107,106].

It is essential to emphasize that the mechanistic evidence linking β5i activity to macrophage-driven chronic inflammation and tissue remodeling derives primarily from sterile non-viral models (ovalbumin allergen or bleomycin challenge) [107,109,110]. Extrapolating these findings to severe viral disease suggests that post-viral pulmonary fibrosis, a critical sequela in patients recovering from severe IAV pneumonia, is driven by a self-sustaining pro-fibrotic surge in recruited bone-marrow-derived alveolar macrophages [107,109]. Because β5i deficiency impairs the recruitment and pro-fibrotic signaling (TGF-β1) of these macrophages in non-viral models, targeting β5i represents a potential therapeutic avenue to prevent long-term fibrotic remodeling following severe influenza [107–109,115]. The divergent roles of iP across distinct pulmonary compartments are illustrated in Figure 3.

The spatiotemporal mismatch of iP activity determines the trajectory of lung disease [10,11,68]. As discussed in Section 5.1, during acute IAV infection, rapid iP induction is required early in epithelial cells (0-48 h) to facilitate antigen presentation, establish an antigenic shield, and facilitate clearance [11,19]. However, at later stages (72h+), persistent iP activity in infiltrating myeloid cells can exacerbate tissue damage [104,105]. This suggests that global proteasome inhibitors (like Bortezomib) often fail due to their inability to account for this temporal specificity [105,116]. Effective therapeutic strategies for ARDS or pulmonary fibrosis, must therefore prioritize cell-type specific modulation, focusing on suppressing the pro-fibrotic surge in recruited macrophages while preserving the essential degradative and signaling functions of the alveolar epithelium [117].

While these findings provide a valuable conceptual framework, they must not be interpreted as direct evidence for IAV pathogenesis. In severe influenza, although fibroproliferative repair and ARDS represent major clinical complications [104,105], whether sustained macrophage iP activation is the primary driver of post-viral fibrosis requires direct testing in viral challenge models. Applying β5i inhibitors to prevent post-influenza remodeling therefore remains a promising hypothesis extrapolated from sterile paradigms rather than a clinically or virally validated mechanism (Figure 3) [107,115].

### 5.3. The inflammaging connection

Older adults suffer disproportionately high morbidity and mortality from IAV infection [118]. While systemic baseline inflammation is elevated in the elderly, a state known as inflammaging, the inducible proteasomal switch required for acute viral defense is severely blunted [119]. Understanding the molecular mechanisms driving this age-related deficit is critical for improving clinical outcomes in vulnerable populations. Research in human nasal and bronchial epithelial cells from aged donors demonstrates a marked reduction in the induction of iP following IFN stimulation [103,119]. Mechanistically, this blunted response stems from age-dependent decay in type I and III IFN receptor signaling, characterized by reduced STAT1 phosphorylation and nuclear translocation [103]. Furthermore, age-associated epigenetic remodeling, specifically increased histone deacetylation and promoter hypermethylation at PSMB loci, restricts chromatin accessibility, preventing rapid *de novo* transcription during viral sensing [119]. The inability to rapidly induce iP assembly creates a severe defect in adaptive immune activation. In aged DCs and respiratory epithelial cells, the delayed transition from cP to iP results in defective processing of viral DRiPs. Consequently, the density of surface MHC class I complexes loading immunodominant viral epitopes (such as M1_58-66_ and NP_366-374_) is significantly diminished, leading to delayed CD8^+^ CTLs recruitment and impaired viral clearance [103,119]. Beyond immune evasion, blunted iP induction leaves the senescent respiratory epithelium vulnerable to proteotoxic stress. Aging lungs exhibit higher basal levels of carbonylated and poly-oxidized proteins [119]. During high-speed IAV replication, the failure of inducible 20S cores to rapidly clear oxidant-damaged proteins leads to the accumulation of toxic protein aggresomes. This proteostatic collapse triggers UPR-mediated apoptosis in type I and II AECs, driving extensive alveolar desquamation, capillary leakage, and lethal ARDS [119,120]. This state of biochemical senescence, characterized by high basal cytokine release coupled with a blunted antiviral proteasomal switch, explains why traditional high dose influenza vaccines often yield sub-optimal CD8^+^ T cell responses in the elderly [103]. From a clinical standpoint, the decay in iP induction kinetics complicates traditional vaccination strategies, which rely on efficient antigen processing to generate a robust adaptive response [111,121]. Therapeutic strategies aimed at restoring STAT1 activation or epigenetically reactivating PSMB expression represent promising avenues to restore the proteasomal shield in aged lungs [103,119]. Thus, future research should focus on therapeutic interventions designed to restore the proteasomal switch in the elderly. Interventions that bypass the attenuated IFN-signaling pathway to directly upregulate iP assembly may restore viral surveillance and improve the resolution of infection without exacerbating the existing state of sterile inflammaging.

### 5.4. Cell-type-specific immunoproteasome dynamics in the infected lung

Rather than operating as a uniform process across the respiratory tract, iP executes distinct functional programs depending on the specific cell type within the pulmonary microenvironment. As the primary targets of IAV entry and replication, non-immune respiratory epithelial cells maintain low baseline iP expression that is rapidly induced by type I and III IFN [67]. In these cells, iP functions primarily as a proteostatic shield, rapidly turning over misfolded viral DRiPs and oxidant-damaged host proteins to preserve cellular barrier integrity. Furthermore, epithelial iP induction drives direct MHC class I presentation, marking infected cells for CD8^+^ CTLs-mediated clearance [67]. In professional APCs, iP subunits are expressed at high baseline levels. In DCs, iP is essential for cross-presentation, processing internalized viral antigens into high-affinity peptides to prime naive CD8^+^ T cells in draining lymph nodes. In resident and recruited AMs, hyper-activation of iP drives canonical NF-κB signaling, orchestrating the surge of pro-inflammatory cytokines (IL-1β, TNF, IL-6). Endothelial cells respond to circulating inflammatory cytokines by upregulating β5i-containing cores. iP activity regulates the expression of cell adhesion molecules (ICAM-1, VCAM-1) and tight junction stability, directly modulating neutrophil extravasation and alveolar-capillary fluid leakage during acute lung injury [122]. In stromal fibroblasts, persistent iP activation promotes myofibroblast differentiation and TGF-β1-driven extracellular matrix deposition, linking post-viral tissue repair to fibrotic remodeling. Cell-type-specific iP functions govern respiratory epithelial proteostasis, myeloid antigen cross-presentation and cytokine surges, and endothelial vascular leakage (Figure 3).

## 6. Therapeutic opportunities

### 6.1. Selective modulation: beyond global inhibition

The therapeutic landscape of the UPS began with non-selective inhibitors like Bortezomib, which targets the β5 (cP) and β5i (iP) subunits but causes significant off-target toxicities. While effective for multiple myeloma, its non-selectivity causes peripheral neuropathy in 30% of patients [116,123]. Second-generation drugs like Carfilzomib and Ixazomib improved efficacy but still lacked subunit specificity [124]. Recent efforts have focused on PR-957 (ONX-0914), a selective β5i inhibitor that exploits the unique S1 pocket of iP (Figure 2) (Table 4) (Table 5). In preclinical models, PR-957 reduces the release of pro-inflammatory cytokines (IFN-γ, IL-2, IL-23) and ameliorates autoimmune pathologies, thereby demonstrating a clear *in vivo* proof-of-concept in preclinical rodent models of severe pulmonary inflammation and acute lung injury/ARDS [107,125,126]. Newer analogs like KZR-329 (β5i-selective) and KZR-504 (β1i-selective) show even greater promise in reducing apoptosis and inflammatory signaling in transplant models [111]. By contrast, small-molecule iP activators remain largely experimental tools in early-stage discovery; no clinically approved iP activators currently exist, and their therapeutic utility in viral infection remains theoretical [111] (Table 5). Crucially, while inhibition is the standard approach for inflammation, activation of iP is an emerging strategy for infection and oncology. Using selective activators to increase MHC-I neo-antigen presentation could enhance CD8^+^ T cell activity, though a balanced approach is required to avoid triggering immunopathology [107,127]. While primarily developed for autoimmunity, these inhibitors offer a potential pharmacological brake for the hyper-inflammation seen in lethal influenza.

**Table 4.** Immunoproteasome′s role with targeted inhibitors, and associated outcomes across inflammatory and infectious diseases.

Disease category/condition	Model type	Role of immunoproteasome	Drugs	Target	Side effects	References
Influenza A Virus	Viral Respiratory Infection	Anti-viral immunity, crucial for degrading viral proteins into peptides for MHC class I presentation	ONX-0914	β1i/LMP2 β5i/LMP7	Early inhibition impairs viral clearance; increased weight loss, mortality, and inflammation in the liver and lungs	[9,10]
ARDS/Acute lung injury	Sterile/Endotoxin Models (LPS, mechanical ventilation)	Amplifies cytokine storm, neutrophil influx, vascular leak	ONX-0914	β5i/LMP7	Dampens NF-κB; reduces alveolar-capillary barrier leak; suppression of type I IFN responses, leading to increased viral load and mild myocardial injury	[105–107,137]
COPD/Cigarette smoke	Chronic Environmental/ Sterile Model	Promotes differentiation of pro-inflammatory T helper type 1 (Th1) and 17 (Th17) cells while suppressing the induction of regulatory T cells	ONX-0914	β1i/LMP2 β5i/LMP7	Mitigates macrophage-driven inflammation; potential liver/muscle toxicity	[122,128]
Asthma	Sterile Allergen Model (Ovalbumin/OVA challenge)	Regulates Th2/Th17 cytokine release and airway hyperreactivity	ONX-0914	β1i/LMP2 β5i/LMP7	Attenuates eosinophilic/neutrophilic inflammation and hyperreactivity	[110,138]
SARS-CoV-2/Coronaviruses	Viral Respiratory Infection (MHV-1 animal model)	Pro-inflammatory signaling mediator; antiviral restriction	ONX-0914 Ixazomib	β5i/LMP7	Reduced alveolar inflammation in MHV-1; potential thrombocytopenia with Ixazomib	[26,134]
Pulmonary fibrosis	Sterile Fibrotic Challenge (Bleomycin model)	Drives alternative macrophage polarization and TGF-β1 deposition	ONX-0914	β5i/LMP7	Reduces myofibroblast differentiation and collagen accumulation	[107–109]

**Table 5.** Pharmacological modulators targeting the immunoproteasome.

Compound	Class/Mechanism	Target subunit	Development stage	Primary biological effect	Translational limitations/risks	References
iP activators	Small-molecule allosteric modulators	20S core particle gate opening	Early Discovery (Experimental)	Accelerates peptide entry; theoretically jumpstarts antigen processing	No clinical activators available; Experimental/Unvalidated in viral infection; risk of premature cytokinemia and epitope over-digestion	[107,127]
Ixazomib	Boronic acid derivative (Inhibitor)	Dual β5/β5i	FDA-approved (Oncology)	Broad proteasome inhibition; suppresses hyper-active inflammatory responses	Non-selective β5 target engagement; gastrointestinal toxicity and thrombocytopenia	[124,134]
KZR-329	Selective peptide epoxyketone (Inhibitor)	β5i	Clinical (Phase I/II)	Potent inhibition of cytokine release in inflammatory and autoimmune models	Risk of impairing baseline T-cell epitope presentation during early viral infection	[111]
KZR-504	Selective inhibitor	β1i	Preclinical	Modulates specific cleavage preferences without altering total chymotrypsin activity	Narrow therapeutic window; requires precise timing during inflammatory cascades	[111]
PR-957 (ONX-0914)	Epoxyketone tripeptide (Inhibitor)	β5i	Preclinical	Blocks pro-inflammatory cytokines; dampens NF-κB; reduces acute lung injury	Potential off-target liver toxicity at high doses; unvalidated in acute viral challenge	[125,126]

### 6.2. The timing paradox: a biphasic clinical framework

#### 6.2.1. *In vivo* validated strategies

Direct *in vivo* preclinical evidence demonstrates that late-stage administration (72 hpi) of selective β5i inhibitors (like ONX-0914) significantly reduces alveolar hyper-inflammation, neutrophil recruitment, and vascular leakage in murine models of acute lung injury [26,125,126]. Importantly, while these *in vivo* data confirm that β5i inhibition can alleviate immune-mediated pulmonary injury during peak inflammation, direct preclinical trials of ONX-0914 specifically in severe IAV-induced ARDS models remain limited. In this late phase, viral replication has often peaked, and disease pathogenesis is driven primarily by an overexuberant host immune response. Consequently, suppressing iP-mediated NF-κB activation during peak tissue injury could serve as an evidence-based pharmacological emergency brake but warrants dedicated *in vivo* validation in lethal influenza cohorts.

#### 6.2.2. A hypothesis-generating biphasic framework and its translational realities

To conceptualize how the opposing antiviral and inflammatory actions of the iP might theoretically be managed, we put forward a hypothesis-generating framework rather than a validated therapeutic strategy (Figure 2 and Figure 3). In this conceptual model, intervention during early viral replication would theoretically aim to preserve or accelerate functional iP assembly to overcome viral host shutoff, whereas late-stage intervention would deploy selective inhibitors to dampen destructive hyper-inflammation. We explicitly underscore that early-stage iP activation remains entirely unvalidated and hypothetical in acute viral infections [111]. Multiple formidable biological and translational barriers caution against viewing this as an established clinical paradigm. Firstly, no selective small molecule iP activators have been approved for human use or validated in *in vivo* IAV infection models [111]. Secondly, forced early iP activation carries significant risks of accelerating systemic cytokinemia or causing the proteolytic over-digestion and destruction of vital immunodominant viral epitopes, paradoxically impairing CD8^+^ T-cell priming [11,76,127]. Finally, rigid chronological hour thresholds (0–48 h vs. 72h+) are conceptual simplifications that fail to capture the asynchronous infection kinetics and biological heterogeneity of human respiratory disease. Pinpointing the exact biological transition point from protective host defense to tissue-destructive immunopathology in individual patients remains an unresolved clinical challenge. Consequently, while post-peak selective β5i inhibition is supported by preclinical *in vivo* proof-of-concept in animal models of acute respiratory inflammation, the overarching biphasic framework serves solely as a conceptual basis for future timing-controlled investigations rather than an actionable therapeutic regimen [125,126].

### 6.3. Repurposing geroprotectors to restore the antiviral proteasomal switch in aging

Since advanced age severely blunts iP expression during acute IAV infection (Section 5.3) [103,119], targeted geroprotective strategies represent an appealing avenue to restore host antiviral surveillance in older adults [111,107]. Aged respiratory epithelial cells exhibit impaired STAT1 phosphorylation, delaying *de novo* 20S iP assembly during early viral sensing [103]. Repurposed metabolic and signaling modulators, such as metformin or low-dose JAK/STAT pathway tuners, have been shown to enhance STAT1 responsiveness and reduce basal proteotoxic stress, partially rescuing early iP induction upon viral challenge [103,128]. Further, age-associated histone deacetylation and promoter hypermethylation restrict chromatin accessibility at β1i and β5i loci [119]. Low-dose histone deacetylase inhibitors and epigenetic remodellers can reverse this promoter silencing, enhancing the rate of iP biogenesis during early IAV infection to overcome viral host shutoff [111,119]. By directly targeting the molecular blockades that impair the proteasomal switch in aging lungs, these repurposed strategies aim to bolster early proteostatic buffering and antigen presentation without inducing systemic toxicity [103,107,129,130].

### 6.4. Challenges: the goldilocks balance in IAV therapy

The primary hurdle in targeting iP for IAV is balancing viral clearance with the prevention of immunopathology. In the context of IAV infection, the case for robust iP activity is defined by the absolute necessity of rapid viral clearance to prevent systemic spread. Unlike cP, iP is specialized in processing internal viral proteins such as M1, NP, and PB1 into high-affinity peptides that fit the specific binding requirements of MHC-I molecules [9]. This proteasomal switch ensures that CD8^+^ CTLs can identify and destroy infected respiratory epithelial cells before they become factories for viral progeny. Furthermore, iP is essential for the polyfunctional maturation of these T cells; CTLs primed in an iP-rich environment are more likely to simultaneously produce IFN-γ, IL-2, and TNF, a triple-threat cytokine profile that correlates with superior survival outcomes [131,132]. Balancing viral clearance against immunopathology presents a timing paradox (Figure 2). Rather than repeating the mechanistic details of NF-κB amplification (Section 5.1) or the biphasic therapeutic framework (Section 6.2), the clinical challenge reduces to understanding the kinetics of intervention, while late-stage β5i inhibition (72h+) represents an evidence-supported preclinical strategy to limit acute lung injury [107,125,126], the concept of early-stage activation remains an unproven, theoretical hypothesis that has not been empirically demonstrated in viral infections [111,133]. Achieving the goldilocks balance, preserving sufficient proteasomal activity for viral clearance while dampening the hyper-active cascades that drive tissue damage and ARDS, remains a hypothesis-generating goal that requires navigating complex clinical trade-offs. While selective β5i inhibitors demonstrate clear preclinical *in vivo* efficacy in mitigating hyper-inflammatory tissue injury [125,126], human clinical data in viral infections remain virtually non-existent. Future translational efforts must prioritize identifying accessible biomarkers (such as circulating cytokine ratios or airway iP subunit levels) to accurately guide the narrow window between early antiviral defense and late anti-inflammatory intervention.

## 7. Conclusion: the goldilocks paradigm of proteasomal control

The transition from cP to iP is one of the most profound proteomic reorganizations a cell can undergo. While traditionally viewed through the narrow lens, of MHC-I loading, we now recognize iP as a master proteostatic buffer. Beyond generating viral epitopes, it prevents the catastrophic accumulation of polyubiquitinated aggregates and oxidant-damaged factors that threaten cellular integrity during peak viral replication.

### 7.1. Synthesis: the double-edged sword of viral fitness

The systemic importance of this machinery is best illustrated by iP-deficient models, which show not just altered T-cell hierarchies, but a fundamental failure to maintain cellular integrity under the severe proteotoxic stress [10,11,13,19,68,69]. However, this very efficiency makes iP a prime target for viral sabotage. While direct evidence confirms that the viral NP manipulates constitutive β5 to stabilize pro-viral cascades, the broader concept of proteasomal subversion, formulated here as the molecular filter hypothesis, remains an unproven working model (Section 1.3.3). The putative model hypothesizes that IAV may exploit the altered proteolytic landscape to selectively stabilize replication machinery while accelerating the degradation of antiviral signaling proteins, opening a new frontier in subversion research. Identifying the biochemical signatures that allow viral proteins like NP or NS1 to navigate this switch is critical to our understanding of viral fitness. Testing this hypothesis will require high-resolution quantitative degradomics in iP-knockout models to directly evaluate whether viral polymerases possess intrinsic sequence-specific resistance to β5i-mediated cleavage relative to host restriction factors. Future research must utilize high-resolution proteomics to distinguish whether IAV subversion results from global iP suppression *via* NS1 and PA-X or a more tactical, sequence-specific resistance to β5i-mediated cleavage. This would help us rigorously distinguish the experimentally verified processes from the speculative proposition of an iP-wide molecular filter.

### 7.2. The intermediate mystery

As introduced in Section 2.6, intermediate proteasomes representing transitional hybrid states play a prominent role during early IFN responses. The formation of intermediate hybrids serves as a temporal bottleneck in the establishment of a robust adaptive immune response. Future studies must utilize high-resolution proteomics to determine whether viral proteins like NP possess a sequence-specific resistance to β5i cleavage, allowing them to remain stable in an iP-dominant environment that targets host restriction factors for degradation. Future quantitative degradomics studies must determine whether viral shutoff mechanisms (such as NS1/PA-X) selectively freeze proteasome maturation at this intermediate stage to alter epitope presentation hierarchies.

### 7.3. The future of host-directed therapy: kinetic tuning

Moving forward, we must transition from global proteasome inhibition to kinetic tuning. Broad inhibitors have failed clinically due to toxicity, but selective β1i or β5i modulators offer a sophisticated therapeutic window. Success depends on shifting the focus toward enhancing host-mediated clearance and restorative pathways [112], rather than relying solely on pharmacological signaling blockers. Furthermore, future translational inquiries should evaluate the following hypothesis-generating biphasic framework for timing-dependent modulation of iP (discussed in Section 6.2.2):

a) Early Disease Stage (Hypothetical Concept): Under this working hypothesis, future experimental approaches could aim to preserve or accelerate iP biogenesis to outpace viral shutoff effectors and sustain early CD8^+^ T cell priming. However, this concept remains unvalidated *in vivo* and must be approached with caution due to the absence of targeted activators and the severe risks of exacerbating premature tissue inflammation. Potential strategies include iP-activators or adeno-associated virus-mediated restoration of β5 expression to dampen BIK levels and reduce viral loads [20].
b) Late Disease Stage (Preclinically Supported Concept): During peak inflammatory injury and cytokine surge, selective β5i inhibitors (such as ONX-0914) act as a pharmacological emergency brake to mitigate alveolar destruction and viral ARDS without the broad toxicities of pan-proteasome inhibitors.

In conclusion, the goal is to achieve a goldilocks balance: preserving enough proteasomal activity for viral clearance while dampening the excess that drives tissue damage. Integrating system-level protein turnover data with traditional immunological frameworks will be essential to developing therapies that protect the host from both the virus and itself.

### 7.4. Current knowledge gaps and outstanding questions

To advance the field from conceptual frameworks to definitive mechanistic understanding, several critical experimental gaps must be addressed:

1. Validation of the molecular filter: High-resolution spatial transcriptomics with mass spectrometry-based degradomics are required to definitively establish whether iP preferentially degrades host antiviral sensors (e.g. MAVS, RIG-I) while sparing viral polymerase subunits (PB1, PB2, PA).
2. Experimental validation of the putative kinetic lag in primary cells: While cytokine-driven iP transcriptional induction is well documented, direct empirical evidence for impaired functional 20S core particle maturation during early IAV infection in primary human AECs remains limited. Quantitative pulse-chase, native gel, and degradomics analyses in primary polarized human AECs are required to definitively test whether viral shutoff translates into a physiologically meaningful kinetic delay in mature iP activity.
3. Validation of chaperone competition: Experimental testing is required to determine whether ER-resident folding stress induced by HA/NA directly impacts cytosolic/nuclear POMP availability or 20S maturation rates.
4. Direct *in vivo* validation of immunoproteasome-driven pathogenesis in IAV vs. sterile models: While β5i-dependent macrophage polarization and TGF-β1-driven fibrotic signaling are well documented in sterile bleomycin and allergen challenge models, direct experimental proof that persistent iP activation drives macrophage-mediated cytokine storms and long-term fibrotic remodeling specifically during IAV pathogenesis is still required. Controlled viral challenge studies comparing wild-type and β5i^-/-^ mice during late-stage infection and recovery are necessary to separate IAV-specific pathology from general sterile tissue injury responses.
5. Dissecting iP-specific proteostasis from general UPS and autophagic flux: While the UPS, ERAD, and autophagy collectively manage viral protein load and oxidant stress during IAV infection, the specific, non-redundant contribution of inducible iP subunits in buffering epithelial proteotoxicity remains unproven. Future studies employing subunit-selective inhibitors or genetic knockouts alongside autophagic blockers are required to determine whether iP provides distinct proteostatic advantages in infected airway epithelium.
